# Towards Genomic Criteria for Delineating Fungal Species

**DOI:** 10.3390/jof6040246

**Published:** 2020-10-24

**Authors:** Cene Gostinčar

**Affiliations:** 1 Department of Biology, Biotechnical Faculty, University of Ljubljana, Jamnikarjeva 101, SI-1000 Ljubljana, Slovenia; cene.gostincar@bf.uni-lj.si; Tel.: +386-1-3203405; 2 Lars Bolund Institute of Regenerative Medicine, BGI-Qingdao, Qingdao 266555, China

**Keywords:** fungi, species delineation, species concept, genomic distance, species similarity, mash, dashing

## Abstract

The discussion of fungal species delineation has yet to reach a consensus, despite the advancements in technology, which helped modernise traditional approaches. In particular, the phylogenetic species concept was one of the tools that has been used with considerable success across the fungal kingdom. The fast rise of fungal genomics provides an unprecedented opportunity to expand measuring the relatedness of fungal strains to the level of whole genomes. However, the use of genomic information in taxonomy has only just begun, and few methodological guidelines have been suggested so far. Here, a simple approach of computationally measuring genomic distances and their use as a standard for species delineation is investigated. A fixed threshold genomic distance calculated by the quick and easy-to-use tools Mash and Dashing proved to be an unexpectedly widely applicable and robust criterion for determining whether two genomes belong to the same or to different species. The accuracy of species delineation in an uncurated dataset of GenBank fungal genomes was close to 90%—and exceeded 90% with minimal curation. As expected, the discriminative power of this approach was lower at higher taxonomic ranks, but still significantly larger than zero. Simple instructions for calculation of a genomic distance between two genomes and species similarity thresholds at different k-mer sizes are suggested. The calculation of genomic distance is identified as a powerful approach for delineating fungal species and is proposed—not as the only criterion—but as an additional tool in the versatile toolbox of fungal taxonomy.

## 1. Introduction

How to define a species is a perennial question of biology. The biological species concept of Ernst Mayr [1] for example, while seminal in practice, turned out to be poorly applicable to major parts of the tree of life. Extreme cases in which this concept fails entirely are asexual organisms. This is not only a problem in prokaryotes, but also in fungi. While the frequency of sexual reproduction in fungi appears to be much higher than was previously thought [2], we are, in many cases, still unable to trigger such reproduction in vitro.

However, even in clonal organisms, species appear to reflect a biological reality rather than an artificial concept. The clones are not distributed over an unstructured continuous spectrum, but mimic biological species by forming cohesive clusters, which maintain their within-cluster similarity over time. This phenomenon can be explained by the formation of ecotypes driven by periodic selection or genetic drift or both [3]. These periodically compress the diversity to near zero, unless the accumulating mutations or recombinations place the organism into a new ecological niche, which forms a new (in most cases monophyletic) ecotype with a different set of selection pressures [3]. This process results in discontinuities in the spectrum of possible diversity, leaving more or less well-separated clusters, which can be seen in phylogenetic trees. To describe these clusters, researchers have used both phenotypic and genetic markers and as the methods develop, so does the practical application of biological classification.

The tools used by taxonomists studying different groups of organisms differ substantially. Microbiologists in particular had to resort to innovative solutions to accommodate for the distinctive qualities of their study object. In bacteria, where morphology provides few clues about the taxonomic placement of the organism, researchers have embraced various measures of genomic distance relatively early on, as an addition to time-consuming phenotypic characterization of pure cultures. The DNA base composition, traditionally expressed as the GC-content, could provide information about the dissimilarity of two organisms, but not about their similarity, since two unrelated organisms often have a similar GC-content [4]. In contrast, whole-genome DNA–DNA hybridisation provided a more definitive picture about similarity. Relatedness of 70% was recommended as the threshold, which two strains should exceed in order to be placed in the same species [5,6]. Due to DNA–DNA hybridisation being a technically demanding and cumbersome method, this standard was later extended by another criterion: 97% of 16S ribosomal DNA (rDNA) gene-sequence identity [7]. In recent years, a third measure is being adopted: average nucleotide identity (ANI) calculated with computational comparison of two genome sequences, where 95–96% ANI generally corresponds to the 70% threshold in DNA–DNA hybridisation [8,9]. ANI is now being used on a large scale to improve the taxonomic assignments of prokaryotic genomes in GenBank and the approach is being expanded to eukaryotic genomes as well [10]. Some authors suggest that phenotypic approaches and DNA–DNA hybridisation could be replaced by a core genome phylogenetic analysis and ANI altogether [11]. While, on the one hand, such an approach would be pragmatic, on the other hand, the valuable knowledge about the phenotypic characters of a microbial culture should not be neglected, both for understanding the biology of the species and its potential uses in biotechnology or other fields.

Compared to bacteria, the much larger morphological diversity of fungi supported the relatively greater role of morphological traits in fungal taxonomy. This led to a greater role of the traditional approaches to classification even after molecular techniques became widely available and applicable. Nevertheless, molecular approaches have been adopted as well, and have been used to great effect. While we now know that a majority of fungi might be capable of sexual reproduction and do so at least occasionally [2], the difficulty of triggering such reproduction in a laboratory setting makes the widespread use of the biological species concept, in mycology at least, impractical if not impossible [12]. Instead, the phylogenetic species concept has been used with great success [13], despite warnings that this can lead to taxonomic inflation [14]. Multilocus sequence typing provided valuable information on the levels of recombination within a certain group of organisms [15] and has helped resolve taxonomically difficult groups [16]. Finally, the use of genotypic traits also allowed for the adoption of the “one fungus—one name” principle, again triggering major taxonomic changes and also efforts to avoid excessive nomenclatural instability [17]. Two criteria are generally used for delineating species with phylogenetic information: genealogical concordance (congruence between genealogies of unlinked loci) and formation of reciprocally monophyletic and well-supported groups—a combined approach that has led to description of hundreds of new species [12]. At the same time, projects such as the “Assembling the Fungal Tree Of Life” (AFToL) provided the molecular basis for understanding the fungal evolution at higher taxonomic levels [18] and after centuries of uncertainty finally started to resolve the fungal tree of life [19].

Even after major advances in high-throughput sequencing technologies, which provided us with drastic increases in sequencing capacity accompanied by a just as drastic reduction in costs, fungal genomics lagged behind the bacterial genomics. Larger and more complex fungal genomes are an important reason for this. Nevertheless, the number of sequenced fungal genomes is now increasing dramatically—by October 2020, the number of assembled fungal genomes deposited in GenBank has already exceeded 7000 and the number of represented species exceeded 2000. Sequencing an average fungal genome is now well within reach of even small mycological laboratories. Despite this, examples of the use of whole genomes in species delineation are rare [20,21,22] and hampered by the lack of clear and simple guidelines on the methods for comparison and criteria for interpretation of the results. Some recent publications attempt to outline the general principles for the use of genomics in fungal classification [12]. In addition to applying the monophyly, concordance, and low shared polymorphism criteria to the genomic level, a proposed fourth approach is based on assumption of the genetic discontinuities that exist between species. As logically follows from this, the distance between individuals from the same species should be smaller than between their distances to individuals from other species [12]. One thing these approaches have in common is that they need to be applied and interpreted with respect to the relative phylogenetic background of each taxon. The distance between individuals (from the same species), for example, is interpreted relatively to their distance to other individuals (from other species).

The question investigated here is more absolute and based upon fewer biological assumptions: Can measuring the genetic distances be used to delineate fungal species using a generally applicable similarity threshold, similar to criteria used for delineating bacterial species, while at the same time supporting rather than contradicting the currently accepted classification of fungi? Such approach may appear simple—even simplistic—but, as shown below, it can be applied surprisingly well to species across the fungal kingdom.

## 2. Materials and Methods

### 2.1. Genome Sequences

The complete fungal genome assemblies were downloaded from the GenBank Genome database on 19th March 2020. The list of available genomes was downloaded as the ‘assembly_summary.txt’ file (ftp://ftp.ncbi.nlm.nih.gov/genomes/genbank/fungi/assembly_summary.txt). From the full GenBank Genome database the downloaded dataset was limited to species with at least three available genomes. For species with more than ten available genomes, only ten genomes were randomly chosen in order to limit the overrepresentation of intensely-sequenced species in the final dataset, which consisted of 1544 genomes. The nucleotide genome assemblies (‘*_genomic.fna.gz’ files) were downloaded and used in the downstream analyses. The files were renamed by replacing their original names with their GenBank accession numbers. The taxonomic lineages of the genomes were obtained from the new GenBank taxdump database (ftp.ncbi.nlm.nih.gov/pub/taxonomy/new_taxdump/new_taxdump.tar.gz). The file ‘rankedlineage.dmp’ was used to annotate the genome list with the same taxonomic ranks for each genome: species, genus, family, order, class, and phylum.

For the manual calculation of shared k-mers and for checking the results obtained on the larger dataset with Mash and Dashing, a smaller dataset of 308 fungal genomes were downloaded from the RefSeq database on 19th March 2020. This smaller dataset was used only where specifically stated.

### 2.2. Calculation of Genomic Distances

Genomic distances between pairs of genomes were calculated with three methods, as summarised in Table 1. The programs used were Mash 2.2.2 [23], Dashing 0.4.0 [24], and Jellyfish 2.2.10 [25].

Mash reduces large sequences to small, representative MinHash sketches, from which it can then rapidly estimate global mutation distances. It estimates the Jaccard index of genomic distance, which is the fraction of shared k-mers, as well as a “Mash distance,” which was used here and is a proxy for Average Nucleotide Identity (ANI) [23]. Dashing uses a similar approach, but replaces the MinHash sketches with HyperLogLog sketches, resulting in improved speed and accuracy [24].

The full calculation of the k-mer overlap between two genomes was implemented in a computationally suboptimal way solely for comparison purposes and is not recommended here for general use, especially not over the superior methods such as those implemented in Mash or Dashing. As documented in Table 1, a list of k-mers in a genome was made by Jellyfish. This was then processed into a sorted list of unique k-mers. The overlap between the k-mer lists of two genomes was calculated, the expected coincidental (random sampling, as in ‘capture-recapture’) overlap was subtracted and the resulting number was expressed as a share of the smaller k-mer list.

All calculations were performed with the k-mer size 16, except for the investigation of the influence of different k-mer lengths and the calculation of the final similarity thresholds, as described below. The k-mer size was selected as a compromise between larger sizes, which provide a better resolution at lower taxonomic levels and smaller sizes, which are more suitable for more divergent genomes. Relatively large sketch sizes were used in mash and dashing to improve the accuracy of the genomic distance estimates, which is important especially for comparing divergent genomes.

Custom scripts written in Bash were used to calculate the distances between all possible pairs within each species, between all possible pairs belonging to different species in the same genus, between all possible pairs belonging to different genera in the same family and so on for all six investigated taxonomic levels. In case of the manual k-mer overlap calculations all genomes were compared only on the species and genus levels, for other levels the genomes were limited to those found in the RefSeq database due to the relatively slow calculation speed and large number of comparisons at higher taxonomic levels. In case of Mash and Dashing, the whole dataset of genomes was used at all taxonomic levels.

For the determination of the final genus-species similarity thresholds (but not for other analyses in the study) a subset of genomes was created from the full GenBank dataset by removing *Saccharomyces* species hybrids, species containing strain numbers instead of species names, and the most extreme outliers—genera with unexpectedly short between-species distances (*Komagataella*, *Calonectria*, *Coccidioides*, and *Alternaria*) and species with unexpectedly high within-species diversity (*Rhizoctonia solani, Debaryomyces hansenii, Blumeria graminis, Parastagonospora avenae, Schyzophyllum commune, Rhodotorula toruloides, Hortaea werneckii,* and *Microbotryum violaceum*). This reduced dataset contained 1319 genomes (85% of the initial dataset).

The scripts used in the study are provided in File S3.

### 2.3. Statistics

To check for statistically significant differences between genomic distances at different taxonomic levels, ANOVA with type III sum of squares was calculated with the function ‘Anova()’ provided in the R package ‘car’ [26,27].

Post-hoc pairwise estimated marginal means were calculated between all possible pairs of different taxonomic levels with the function ‘emmeans()’ provided in the R package ‘emmeans’ [26,28].

The thresholds between different taxonomic levels were calculated in R using a custom binary search algorithm to investigate the space between the median genomic distance values of the compared taxonomic levels until both compared taxonomic levels contained a nearly identical share of genome pairs on the wrong side of the threshold (with difference < 0.0001) or until the search completed 100 iterations.

All visualisations were made in R using packages ‘dplyr’ and ‘ggplot2’ [26,29,30].

### 2.4. Computational Power

All in silico analyses were performed on a workstation with the AMD Ryzen Threadripper 1950X processor, 128 GB DDR4 ECC random-access memory and 1 TB Samsung 960 NVMe SSD PRO disk and in a Linux operating system. The Linux distribution used was Ubuntu MATE 18.04 (https://ubuntu-mate.org/).

## 3. Results

Pairwise genomic distances were calculated between 1544 assembled fungal genomes deposited in GenBank at different taxonomic levels (species, genus, family, order, class, and phylum) using Mash and Dashing. A total of 5867 genomic distances were calculated at the species level, 19,849 at the genus level, 13,152 at the family, 58,097 at the order, 68,175 at the class, and 566,436 at the phylum levels. The share of overlapping k-mers was calculated for this dataset only at the species and genus levels, while at other levels only the 308 genomes of the RefSeq database were compared.

**Figure 1:**
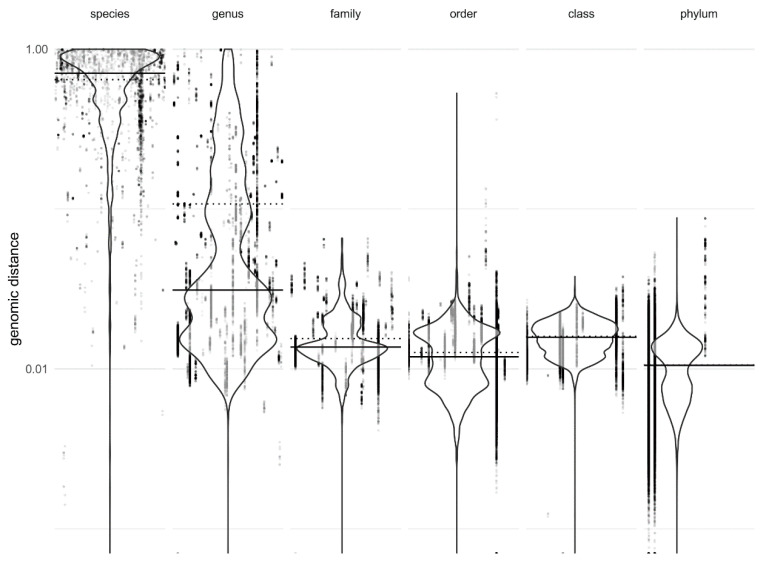
Genomic distances between pairs of fungal genomes at various taxonomic ranks, calculated by Dashing with k-mer size 16. The distances on the species level are between genomes in the same species, the distances on the genus level are distances between genomes of different species belonging to the same genus and so on. Density (violin) plots are plotted on top of points showing intergenomic distances (one point per genome pair). Points representing the same taxon (e.g., distances within the same species on the species level) are arranged in columns. Solid horizontal lines mark the median genomic distances, dashed horizontal lines mark the average genomic distances per taxonomic rank. The *y*-axis is logarithmic.

The differences between the different taxonomic levels (Figure 1, Figures S1 and S2) were statistically significant, as confirmed by the ANOVA, and post-hoc pairwise estimated marginal means showed that significant difference was observed between most pairs of taxonomic levels (Table S1) for both GenBank and RefSeq datasets and for all three used methods of calculating the genomic distance. Dashing could not distinguish between the family and class, while the share of overlapping k-mers could not distinguish between the order and the class as well as between the order and the phylum levels.

**Figure 2:**
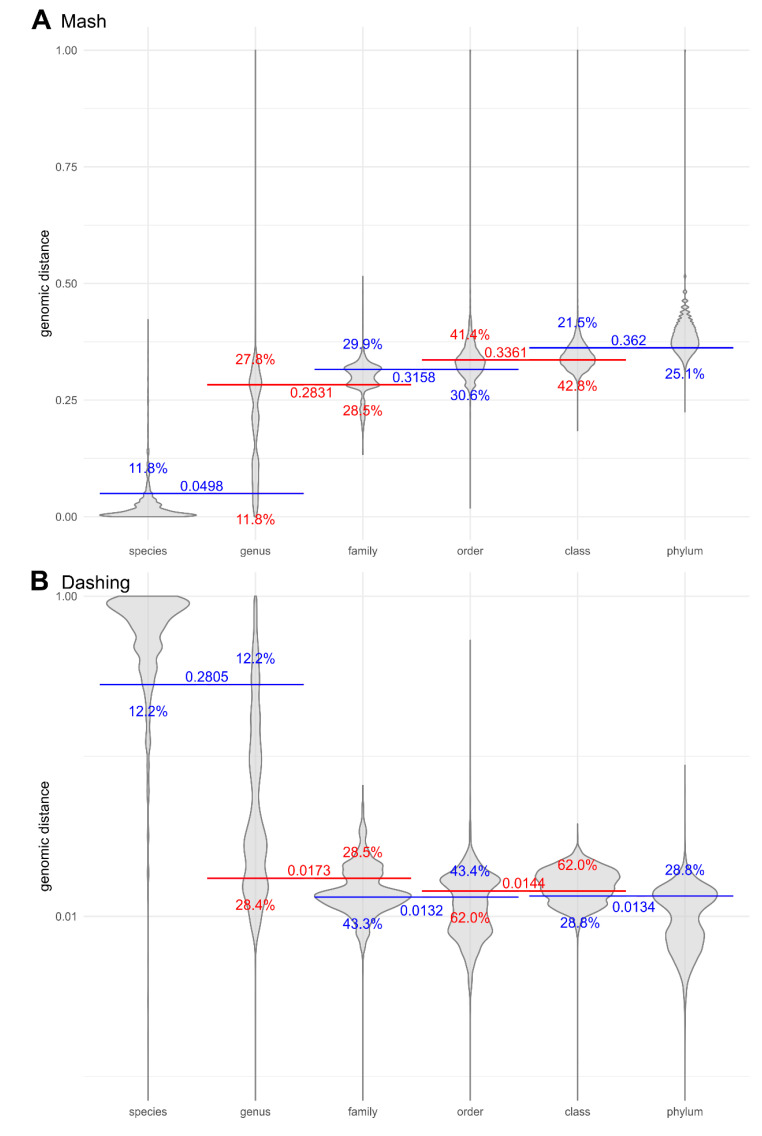
Genomic distances between pairs of fungal genomes at various taxonomic ranks, calculated by (**A**) Mash and (**B**) Dashing with k-mer size 16. Horizontal lines crossing pairs of taxonomic ranks show the best division lines between the ranks, calculated to minimize the share of genomic distances on the wrong side of the line (misclassified distances). Numbers next to the line mark the *y*-axis intercept (the genomic distance threshold), and percentages show the proportion of misclassified distances by horizontal line closest to them. The *y*-axis is linear for Mash distances (**A**) and logarithmic for Dashing distances (**B**).

**Figure 3:**
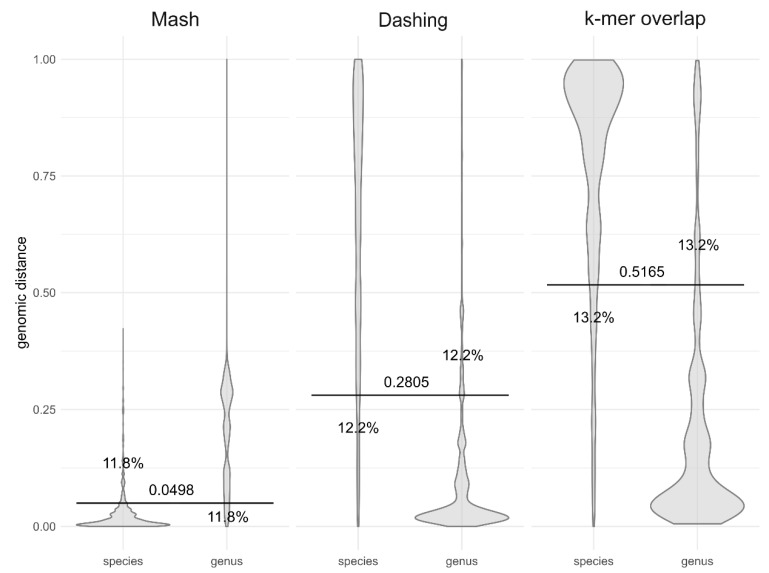
Comparison of different methods of genomic distance calculation in separating the within- and between-species distances. Genomic distances were calculated by Mash, Dashing, and as the share of overlapping k-mers, all with k-mer size 16. Horizontal lines crossing pairs of taxonomic ranks show the best division lines between the ranks, calculated to minimize the share of genomic distances on the wrong side of the line (misclassified distances). Numbers next to the line mark the *y*-axis intercept (the genomic distance threshold), and percentages show the proportion of misclassified distances by horizontal line closest to them.

Threshold genomic distances could be used to distinguish the distances within the species from the distances between the species of the same genus (Figure 2 and Figure 3), so that only 11.8% and 12.2% of distances as calculated by Mash and Dashing, respectively, remained on the other side of the threshold. After removing the *Saccharomyces* species hybrids and species containing strain numbers in the species names, the species-genus classification accuracy with Dashing genomic distances increased to 91.39%. The accuracy of the k-mer overlap measure was slightly less accurate at 13.2% misclassified pairs (Figure 3). The borders between higher taxonomic ranks were less well defined (Figure 2).

**Figure 4:**
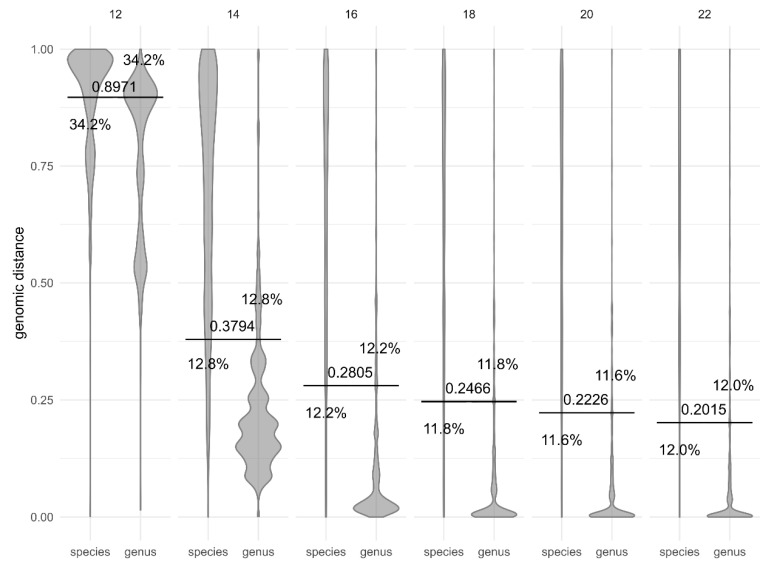
Comparison of different k-mer sizes (12–22, top) used in genomic distance calculation with Dashing in separating the within- and between-species distances. Horizontal lines crossing pairs of taxonomic ranks show the best division lines between the ranks, calculated to minimize the share of genomic distances on the wrong side of the line (misclassified distances). Numbers next to the line mark the *y*-axis intercept (the genomic distance threshold), and percentages show the proportion of misclassified distances by the horizontal line closest to them.

The selection of k-mer length (Figure 4) influenced the absolute threshold value, but with the exception of length 12, the accuracy of species-genus distinction was comparable for all investigated values between 12 and 22, achieving the best accuracy at k-mer length 20 (11.6% wrong classifications). Since shorter k-mers are more sensitive for divergent genomes [23], the somewhat smaller accuracy at k-mer length 16 was deemed an acceptable trade-off for greater sensitivity at higher taxonomic levels. Therefore, all other analyses reported here were performed at this k-mer length.

**Figure 5:**
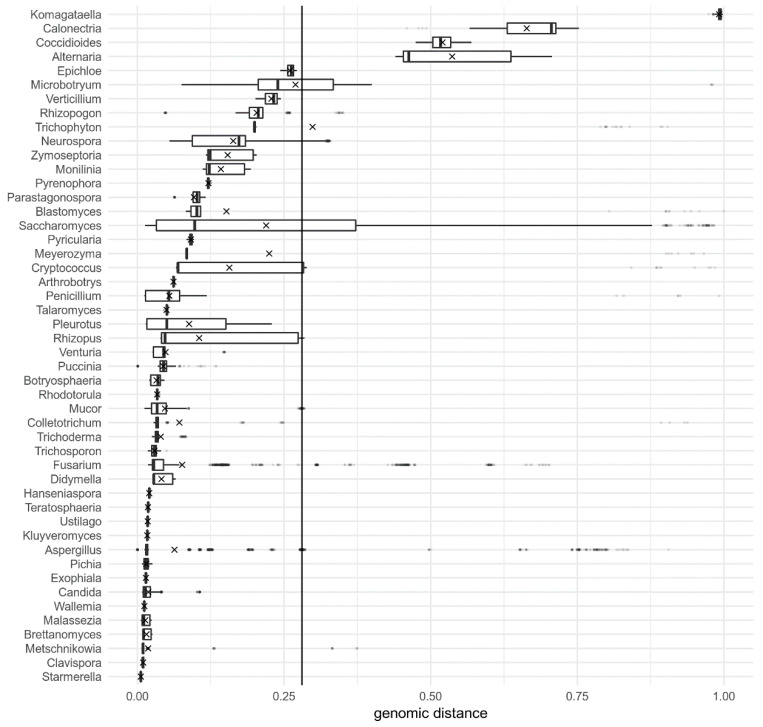
Pairwise genomic distances between all genomes of various genera, calculated by Dashing with k-mer size 16. Distances are shown as box-and-whiskers plots, with outliers marked as dots and mean distances per genus marked with crosses. The solid vertical line shows the threshold distance between the ranks, calculated to minimize the share of genomic distances on the wrong side of the line (misclassified distances). Only genera with at least 20 pairwise distances are shown.

The approximately 12% of genome pairs violating the above determined species similarity threshold of genomic distance calculated by Dashing (given their current taxonomic placement) were not randomly distributed between different genera and species (Figure 5 and Figure S3). The main genera in which between-species distances were smaller than expected were *Komagataella*, *Calonectria*, *Coccidioides*, *Alternaria*, partially also *Microbotryum* and *Saccharomyces*, and to a small extent *Fusarium* and *Aspergillus* (Figure 5). On the other side of the threshold, species with a higher within-species diversity than expected were *Rhizoctonia solani, Debaryomyces hansenii, Blumeria graminis, Parastagonospora avenae, Schyzophyllum commune, Rhodotorula toruloides, Hortaea werneckii, Microbotryum violaceum*, and to some extent *Saccharomyces kudriavzevii, Candida boidinii, Rhizopus microsporus*, and *Aspergillus terreus* (Figure S3). The full list of genome pairs violating the Dashing genus-species threshold determined here is available as File S2.

**Figure 6:**
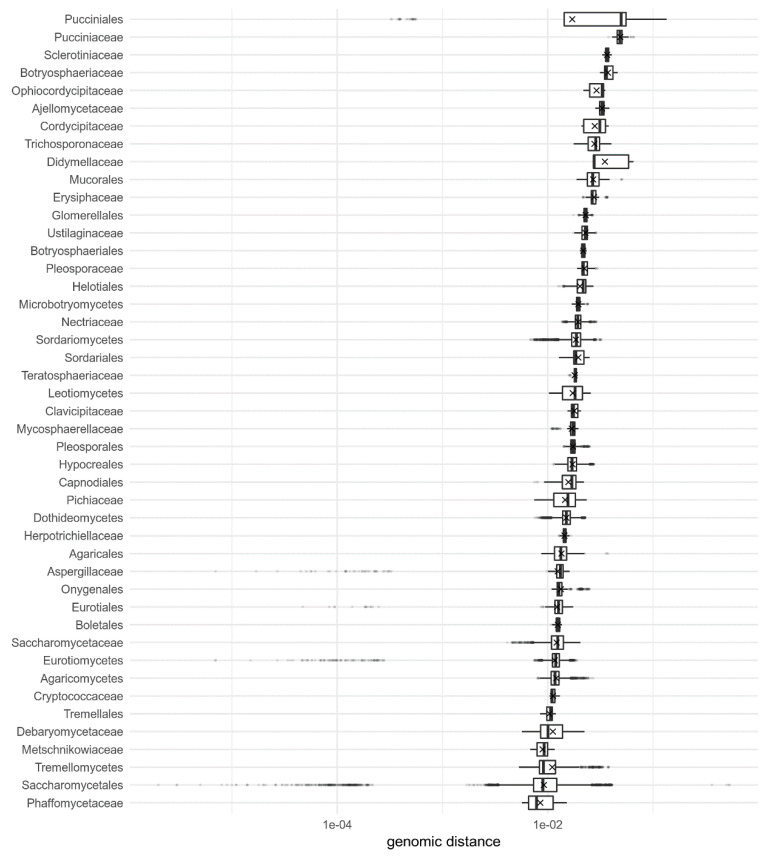
Pairwise genomic distances within fungal families, orders, and classes, calculated by Dashing with k-mer size 16, ordered by their median genomic distances. Distances are shown as box-and-whiskers plots, with outliers marked as dots and mean distances per genus marked with crosses. Only genera with at least 20 pairwise distances are shown.

Comparison of genomic distances within families, orders and classes (Figure 6 and Figure S4) shows that there is a fair amount of deviation from their taxonomic ranking. The median distances within the orders Pucciniales, Mucorales, Glomerellales, Botryosphaeriales, and Helotiales for example are smaller than in many fungal families. Similar observation can be made for Microbotryomycetes and Sordariomycetes. At the same time, the median distances within the families Cryptococcaceae, Debaryomycetaceae, Metchnikowiaceae, and Phaffomycetaceae were comparable to the median distances in several fungal classes.

**Figure 7:**
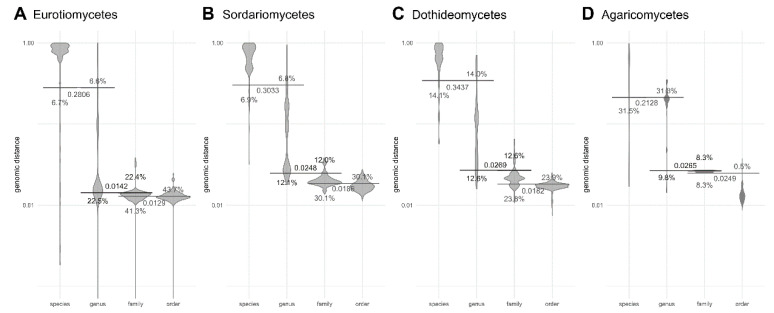
Genomic distances between pairs of fungal genomes at various taxonomic ranks within Eurotiomycetes (**A**), Sordariomycetes (**B**), Dothideomycetes (**C**), and Agaricomycetes (**D**), calculated by Dashing with k-mer size 16. Horizontal lines crossing pairs of taxonomic ranks show the best division lines between the ranks, calculated to minimize the share of genomic distances on the wrong side of the line (misclassified distances). Numbers next to the line mark the *y*-axis intercept (the genomic distance threshold), percent show the proportion of misclassified distances by horizontal line closest to them. The *y*-axis is logarithmic.

However, when looking at certain classes with sufficient numbers of published genomes for the analyses, the distances follow the taxonomic ranks much more clearly (Figure 7). In Sordariomycetes, for example, within-genus genomic distances could be separated from between-genus distances with 88% accuracy, and in Dothideomycetes even the within-family and between-family distances could be separated with 76% accuracy.

The final genus-species thresholds (Table 2) were calculated on a reduced genome dataset of 1319 genomes after removing the incompletely classified strains, *Saccharomyces* hybrids and outlier genera and species. This was done to maximise the quality of the threshold estimations. These thresholds divided the within-species distances from between-species distances with similar accuracy over a wide range of k-mer sizes (14, 16, 18, 20, and 22) with a 0.17% difference between the minimum and maximum accuracy in case of Mash distances and 0.60% difference in accuracy in case of Dashing distances.

## 4. Discussion

The ease of genetic marker sequencing facilitated the use of the phylogenetic species concept in fungal taxonomy [13], especially for fungal groups in which morphological characters are less variable or less informative. Internal transcribed spacers and rDNA genes have become the most popular molecular taxonomic markers, accompanied by the establishment of specialised and often well-curated databases such as UNITE [31] and SILVA [32]. Multilocus sequence typing alleviated some problems of using a single genomic locus and provided some information on the amount of recombination between different lineages [15]. Despite several attempts, these approaches were never fully standardised and there remain large gaps in the sequence databases [33]. The AFToL project for example settled on the use of eight gene loci [18], but the use of so many loci in a description of a species is not the norm. The combination of two criteria, genealogical concordance and reciprocal monophyly of well-supported groups, was successfully applied over the years. The increasing availability of fungal genomic data has led to suggestions of expanding the above approaches mostly in quantity, i.e., by using a larger number of genomic loci [12]. While the use of phylogenomics greatly advanced the understanding of high-level fungal evolution [34,35,36], for everyday use such expansion in scale is not trivial. Many publicly available genomes are not annotated, and those that are, are often not annotated with comparable methods, which makes even obtaining the sequences of the desired loci from public databases a confusing task for users not savvy in bioinformatics—which is perhaps one of the reasons why this approach has not (yet) been widely adopted. Adding to the confusion is the fact that the popular rDNA markers are typically missing from genomic sequences or are incorrectly assembled [37]. Possible uses of genomic data in fungal taxonomy often suffer from a lack of clear guidelines for their application and interpretation of results or from a high degree of sophistication, demanding expert knowledge of bioinformatics. For these or other reasons many otherwise promising approaches were not used beyond the initial study. Magain et al. [38] suggested to use conserved genomic collinearity as a source of markers to resolve species complexes. Sepúlveda et al. [22] showed cryptic speciation in *Histoplasma* using population genomics. Gostinčar et al. [21] used pairwise Kr genomic distances to illustrate the dissimilarity between newly described species of *Aureobasidium* relative to the distances between selected *Saccharomyces* spp.. The use of average nucleotide identity (ANI) for delineation of bacterial species is increasingly accepted [8,9,11]. While in fungi ANI was used to assess the variability of some taxa (e.g., [39]), its use in species delineation is limited by the lack of investigation of the genomic distance thresholds applicable in mycology [20].

*In silico* calculation of a genomic distance between two genomes is a simple operation. In this study, the possible usefulness of genomic distances for delineation of fungal species was investigated. It was found that in the comparison of genome pairs within and between species of the same genus, these two groups could be correctly distinguished in almost 90% of cases, using an uncurated dataset of fungal genomes from GenBank (and in more than 90% of the cases on a minimally curated dataset). The tools used here for genomic distance calculations are simple to use, they are fast, they have minimal hardware requirements, and they estimate distances, which are proxies for ANI. Mash was released in 2016 and has quickly become one of the standard tools of comparative genomics [23]. Dashing is built upon the same principles, but with modifications that provide a more rapid execution and, according to its authors, more accurate results [24].

As illustrated in Figure 5 and Figure S3, there are few outliers that seriously violate the genus-species genomic distance thresholds that are otherwise accurate for almost 90% of genomes investigated in this study (Table 2). There may be several reasons for the outliers. The first and the simplest is misidentification of the species in GenBank, although the number of true errors may be smaller than generally believed [40]. Also, due to more efforts required to produce a genome sequence compared to sequencing a single locus, genomic sequences likely receive more attention in terms of correct identification and deposition. However, fungal taxonomy is in a state of flux and various databases do not always correctly and promptly reflect the state of the art in the field, especially since the authors of sequences are not obliged to provide updates to their records. Also, the NCBI Taxonomy Database is not always in sync with other taxonomic databases, such as Index Fungorum and MycoBank. For example, the unexpectedly high similarity of *Komagataella phaffii* and *Komagataella pastoris* genomes was the reason for the outlier status of the corresponding genus in Figure 5. Both species are synonymous with *Pichia pastoris* and were split into separate species in 2005 [41]. A closer look revealed that the ‘*K. pastoris’* genomes analysed in this study should actually be classified as *K. phaffii*. The genome distances between correctly identified *K. phaffii* and *K. pastoris* as sequenced by Love et al. [42] were well within the expected range of values listed in Table 2. Small distances within the genus *Coccidioides* are likely an artefact due to the high similarity between *Coccidioides posadasii* and unclassified strains of the same genus possibly belonging to *C. posadasii*, but in the analysis treated as a separate species. Additionally, genomes analysed in this study belong to species from different parts of the fungal tree and it is likely that these species were defined using various species concepts. With this in mind, the robustness of the classification provided by genomic distances is actually unexpectedly high.

The second reason for some taxa being outliers in genomic distances may be in suboptimal taxonomic resolution of certain genera or species. *Rhizoctonia solani*, the most diverse species in the dataset, is actually a species complex [43]. The same is true for *Microbotryum violaceum* [44], with many genomic distances within this species not only larger than expected but also larger than distances between some strains of *M. violaceum* and strains of *Microbotryum lychnidis-dioicae*. Unexpectedly small inter-species distances in the genus *Alternaria* (Figure 5) are a consequence of the high similarity between *Alternaria arborescens*, *Alternaria alternata*, and *Alternaria tenuissima*, three difficult to distinguish and closely related morphospecies of the section *Alternaria*, of which *A. alternata* and *A. tenuissima* have already been synonymised after genome sequencing [45].

The third reason is a real biological background of the outlier status. One fixed measure, even if it is derived from the whole genome, cannot be expected to be universally applicable. After all, different fungi evolve under different selection pressures, have different reproduction and recombination strategies, different ploidies and differ in other biological traits that can influence the divergence of their genomes. This is exemplified by *Hortaea werneckii*, an extremely halotolerant black yeast with a very peculiar genome. The taxonomy of the species was recently carefully revised, but despite the very large within-species genomic distances it could not be broken into several species [46]. The reason for this was that while the population genomics indicates the species is strictly clonal, the diploid genomes of the species are hybrids of relatively divergent haploid genomes and these hybrids appear to be stable over long periods of time [47]. The list of outliers in File S2 is therefore not necessarily a list of genomes with incorrect taxonomic placement or a list of taxonomic groups with unresolved taxonomy, as long as there are good biological reasons for their outlier status. In another example, the unusually high diversity of *Schizophyllum commune* has been explained by an elevated mutation rate and high effective population size [48]. On the other side of the threshold, the criteria proposed here cannot distinguish between *Trichophyton rubrum*, *T. soudanense*, and *T. violaceum*, three dermatophytes known to have very similar genomes and representing entities with incomplete lineage sorting—but nevertheless maintained as separate species due to coherent differences in morphology, physiology, genetics and, perhaps most importantly, different clinical outcomes [49]. The small distances observed within the genus *Calonectria* were due to the high similarity of *Calonectria pseudonaviculata* and *Calonectria henricotiae*; the latter was separated from the former in 2015, but is closely related to it, with no polymorphisms in the ITS region and few differences in other phylogenetic markers [50]. Finally, some of the outliers are simply artefacts produced by the specific way species fields are populated in the GenBank database: as a consequence, “*Cryptococcus gattii* VGI” and “*Cryptococcus gattii* VGII” were treated as different species in the automated analysis and so were “*Neurospora* sp. CHS2018a” and “*Neurospora* sp. CHS2018b” as well as various hybrids of *Saccharomyces* spp. The true classification power of the genomic distance approach is therefore actually greater than it is determined above and after removing these listed genomes from the dataset, the number of genome pairs violating the genus-species threshold was reduced to below 9%.

Although providing genomic distance thresholds to distinguish between the higher taxonomic ranks was not the main aim of this study, statistically significant differences between most of the higher taxonomic levels were observed. This was somewhat unexpected, since higher taxonomic ranks are not based on biological criteria, but are pragmatic categories formed to provide a useful system of classification for a given group of organisms, and are therefore expected to vary substantially between different parts of the fungal tree of life. Furthermore, the accuracy of the sketch-based methods of calculating the genomic distances deteriorates with growing divergence, even though this effect can be mitigated by increasing the sketch size [23,24]. Nevertheless, the threshold between the order and the family and also between the class and the phylum could still be determined with over 70% accuracy (Figure 2). Genomic distances calculated by Mash better followed the hierarchy of the higher taxonomic levels than distances calculated by Dashing (Figure 1 and Figure S1), despite the reported greater accuracy across a wide range of input sizes and sketch sizes reported for Dashing [24]. When looking at isolated fungal classes, in some (but not all) of them the taxonomic ranks could be delineated with even greater precision (Figure 7). The latter is less surprising: if the class is a monophyletic group of monophyletic orders, the distances between the orders are logically expected to be greater than the distances within the orders. The fact that this assumption is in some classes true also for lower taxonomic ranks—the genera and families in Sordariomycetes and Dothideomycetes could be delineated with almost 90% accuracy—may indicate that the genera and families in these classes are described at similar levels of diversity. It could also be a consequence of limited sampling, although the limitation was not extreme: the Dothideomycetes dataset contained 14 families and 26 genera, the Sordariomycetes dataset contained 16 families and 27 genera.

Two more factors should be considered in the above outlined proposition to use genomic distances as a taxonomic criterion. The first is that while the dataset of genomes used here is considerable, it nevertheless represents only a small part of the vast fungal diversity. As new species are sequenced, the threshold and accuracy estimates of this study may change in ways that are difficult to predict, although major changes would be unexpected. The second is the possibility of using unassembled high-throughput sequencing reads for the calculation of genomic distances—a possibility that was not investigated here. Both Mash and Dashing accept sequencing reads as input [23,24]. Such analysis should be performed with special care due to the presence and potentially high impact of sequencing errors, but if it proves sufficiently accurate, it could remove the need for the genome assembly—a possibility that would make the calculation of genomic distances for fungal species delineation even easier to perform.

The suggested use of genomes (let alone shorter sequences) as type material for species description [51] remains controversial [52]. Nevertheless, this should not discourage fungal taxonomists from using genomics as a tool when it is appropriate to do so, as it has been successfully done by bacteriologists. Development of criteria for the use of genomic sequences for classification and taxonomic purposes in a way that is both informative and accessible to the scientific community will allow us to tap into this vast amount of data and complement the established taxonomic approaches. It is not argued here that genome-based criteria are sufficient for species delineation. Quite the opposite—they should be interpreted in the light of the biology of the studied fungi and should not disregard practical considerations, especially in clinically or otherwise relevant taxa. The decisions about species delineation are not always black-or-white and this is unlikely to change in the future. Cryptic species were detected in *Histoplasma* in a well-executed population genomics study [22], but these (biologically well-justified) species turned out to be difficult to use in routine diagnostics [53]. At the same time, *Trichophyton* taxa with very closely related genomes but different clinical outcomes are maintained as separate species [49]. Genomic criteria, including the genomic distances should thus be interpreted in a wider context or used as an indicator to direct further research. The results described here support precisely this effort, by showing that incorporating genomic criteria will not bring about a taxonomic revolution, but will rather provide additional support to the current taxonomy, diversify the available taxonomic methods, and in some cases highlight groups of fungi, the taxonomy of which might benefit from a careful re-evaluation.

## 5. Conclusions

A pairwise comparison of assembled fungal genomes aligns surprisingly well with the existing fungal taxonomy. By simply calculating the genomic distance among them (after removing incompletely determined and hybrid species from consideration), it is possible to determine whether two strains belong to the same or to different species within the same genus with over 90% accuracy. While genomic distance alone is not suggested here to supplant the established taxonomic approaches or even change how fungal species are defined, it is proposed as a useful and powerful additional tool in the versatile toolbox of fungal taxonomy.

## Figures and Tables

**Table 1:** Methods of genomic distance calculation between nucleotide genome assemblies in files A.fna and B.fna with the k-mer size 16.

Method	Commands	Notes
Mash	# with pre-sketching mash sketch -k 16 -s 100000 A.fna mash sketch -k 16 -s 100000 B.fna mash dist A.fna.msh B.fna.msh # alternatively – without pre-sketching mash dist -k 16 -s 100000 A.fna B.fna	sketches of a certain size and k-mer length need to be calculated only once and can then be reused; sketching onto the disk can optionally be skipped and the distance calculated by a single command; only the distance can be extracted by adding “|cut -f 3” to the last command
Dashing	# with pre-sketching dashing sketch --sketch-size 20 --kmer-length 16 A.fna dashing sketch --sketch-size 20 --kmer-length 16 B.fna dashing cmp --presketched --full-tsv \ A.fna.w.16.spacing.20.hll B.fna.w.16.spacing.20.hll # alternatively – without pre-sketching dashing cmp --sketch-size 20 --kmer-length 16 --full-tsv A.fna B.fna	sketches of a certain size and k-mer length need to be calculated only once and can then be reused; sketching onto the disk can optionally be skipped and the distance calculated by a single command; only the distance can be extracted by adding “|tail -n1|cut -f 2” to the last command
k-mer overlap	jellyfish count -m 16 -s 1000M -t 30 -C A.fna -o A.jelbin jellyfish dump A.jelbin > A.jelly sed ’s/>.*//g’ A.jelly | sed ’/^[[:space:]]*$/d’ > A.edited sort A.edited > A.sorted uniq A.sorted > A.jellykmers # same for genome B KMERSIZE=16 KMERSPACE=$(echo "4^$KMERSIZE" | bc) UKMERA=$(cat A.jellykmers | wc --lines) UKMERB=$(cat B.jellykmers | wc --lines) MINKMER=$(( $UKMERA < $UKMERB ? $UKMERA : $UKMERB )) RANDOVERLAP=$(echo "$UKMERA*$UKMERB/$KMERSPACE" | bc -l) COMKMER=$(comm -12 --nocheck-order A.jellykmers B.jellykmers | wc --lines) RESULT=$(echo "($COMKMER - $RANDOVERLAP) / $MINKMER" | bc -l) echo $RESULT	the method is relatively inefficient and slow—it was used here simply as a proof of concept; calculations could be optimised, e.g., by using multi-threaded sorting and implementation of the method in a faster programming language

**Table 2:** Threshold genomic distances between fungal species and genera for different k-mer sizes calculated with Mash and Dashing programs; commands for calculating the distances are listed in Table 1.

Method	k-mer Size *	Same Species	Genomic Distance Threshold **	Different Species
Mash	14	less than	0.004129	greater than
Mash	16	less than	0.04317	greater than
Mash	18	less than	0.05781	greater than
Mash	20	less than	0.05915	greater than
Mash	22	less than	0.05833	greater than
Dashing	14	greater than	0.9076	less than
Dashing	16	greater than	0.3736	less than
Dashing	18	greater than	0.2437	less than
Dashing	20	greater than	0.2084	less than
Dashing	22	greater than	0.1863	less than

* The accuracy of the classification by thresholds was similar for different k-mer sizes in the table (0.17% difference between the minimum and maximum accuracy in case of Mash distances, and 0.60% difference in accuracy in case of Dashing distances). ** Distances were calculated from a reduced genome dataset of 1319 genomes after excluding incompletely identified species, species hybrids and outlier genomes and species (full description of the dataset is provided in the Methods section), therefore the thresholds in this table are different from the thresholds determined on the full dataset (Figure 2, Figure 3 and Figure 4).

## Supplementary Materials

The following are available online at https://www.mdpi.com/2309-608X/6/4/246/s1, Figure S1: Genomic distances between pairs of fungal genomes at various taxonomic ranks, calculated by Mash (A) and as a share of overlapping k-mers (B), both with k-mer size 16, Figure S2: Genomic distances between pairs of fungal genomes at various taxonomic ranks, expressed as a share of overlapping k-mers with k-mer size 16, Figure S3: Pairwise genomic distances between all genomes of various species, calculated by Dashing with k-mer size 16, Figure S4: Pairwise genomic distances within fungal families, orders and classes, calculated by Mash with k-mer size 16, ordered by their median genomic distances, Table S1: *p*-values for post-hoc pairwise estimated marginal means test between pairwise genomic distances at different taxonomic levels. File S1: contains supplementary figures and tables as described above, File S2: contains a list of genome pairs violating the species similarity thresholds as determined in this study. File S3: contains a list of analyzed genomes and custom Bash scripts used in this study.
