# Supplementary material for: Towards Genomic Criteria for Delineating Fungal Species
Source: J Fungi (Basel). 2020 Oct 24;6(4):246. doi: 10.3390/jof6040246 (PMC7711752; doi:10.3390/jof6040246)
Supplement: Supplementary file 1 [file jof-06-00246-s001.zip › Sup1.pdf]

Supplemental Figure S1. Genomic distances between pairs of fungal genomes at various taxonomic ranks, calculated by Mash (A) and as a share of overlapping k-mers (B), both with k-mer size 16. The distances on the species level are between genomes in the same species, the distances on the genus level are distances between genomes of different species belonging to the same genus and so on. Density (violin) plots are plotted on top of points showing intergenomic distances (one point per genome pair). Points representing the same taxon (e.g. pairwise distances within the same species on the species level) are arranged in columns. Solid horizontal lines mark the median genomic distances, dashed horizontal lines mark the average genomic distances per taxonomic rank. Distances in panel A were calculated on a whole dataset (see Methods for details), panel B were calculated on a limited dataset (RefSeq fungal genomes) with the exception of genus and species levels, where the whole dataset was used.

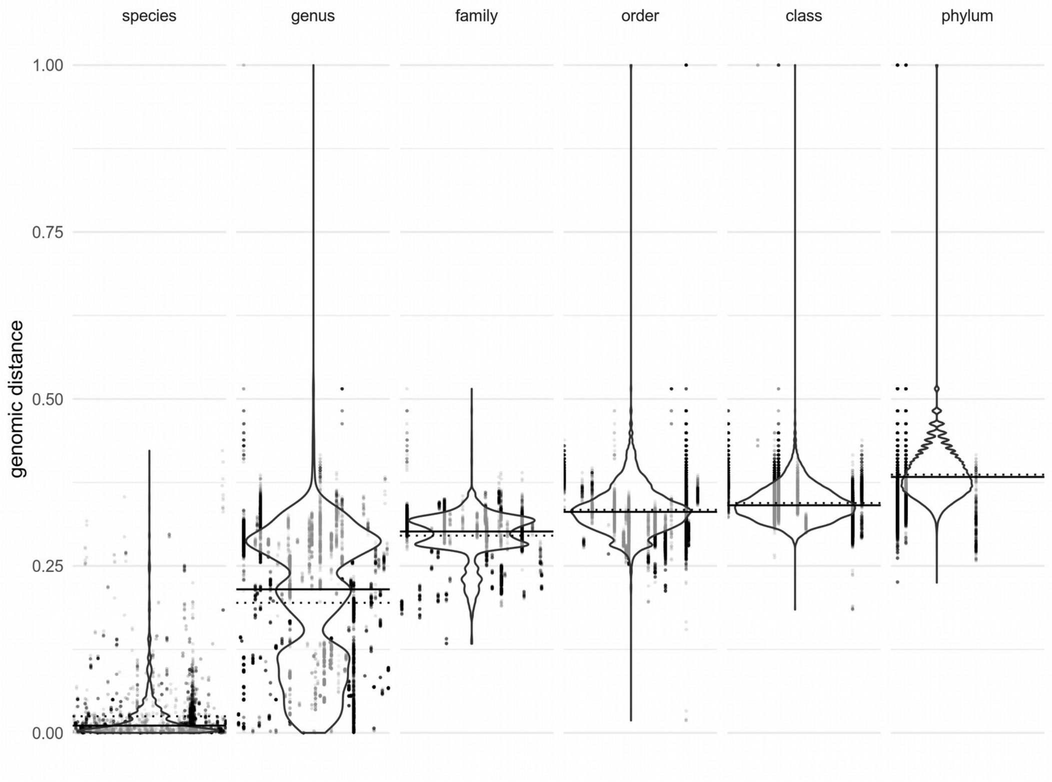

A

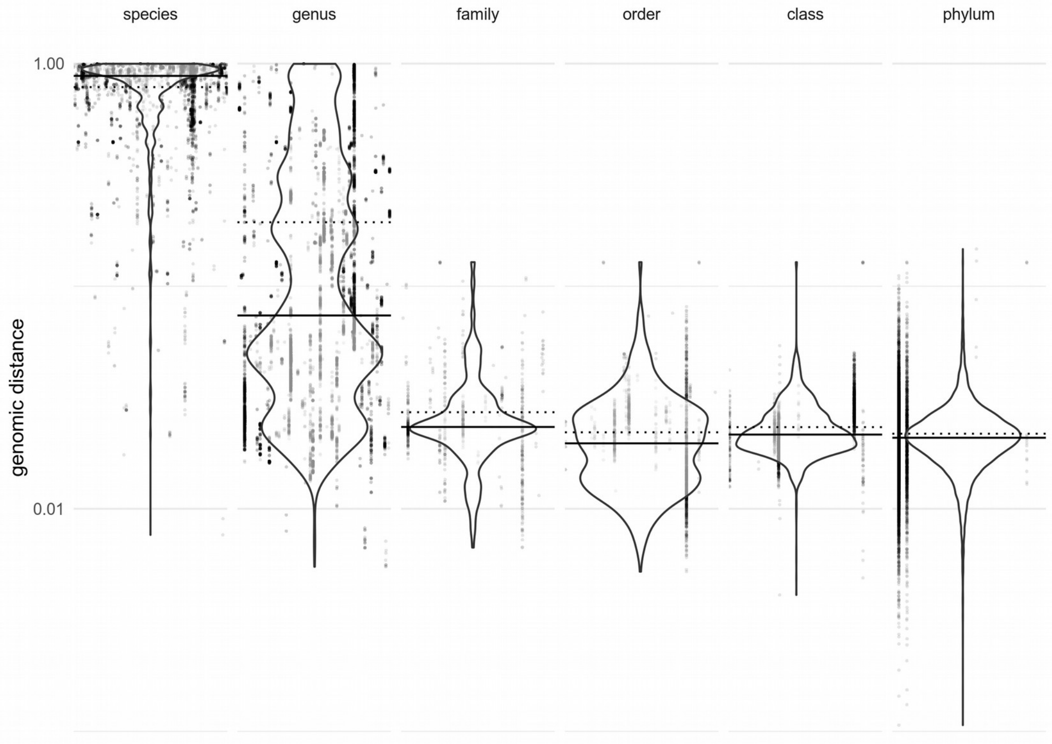

B

Supplemental Figure S2. Genomic distances between pairs of fungal genomes at various taxonomic ranks, expressed as a share of overlapping k-mers with k-mer size 16. Horizontal lines crossing pairs of taxonomic ranks show the best division lines between the ranks, calculated to minimize the share of genomic distances on the wrong side of the line (misclassified distances). Numbers next to the line mark the y-axis intercept (the genomic distance threshold), percent show the proportion of misclasified distances by horizontal line closest to them. The distances were calculated on a limited dataset (RefSeq fungal genomes) with the exception of genus and species levels, where the whole dataset was used.

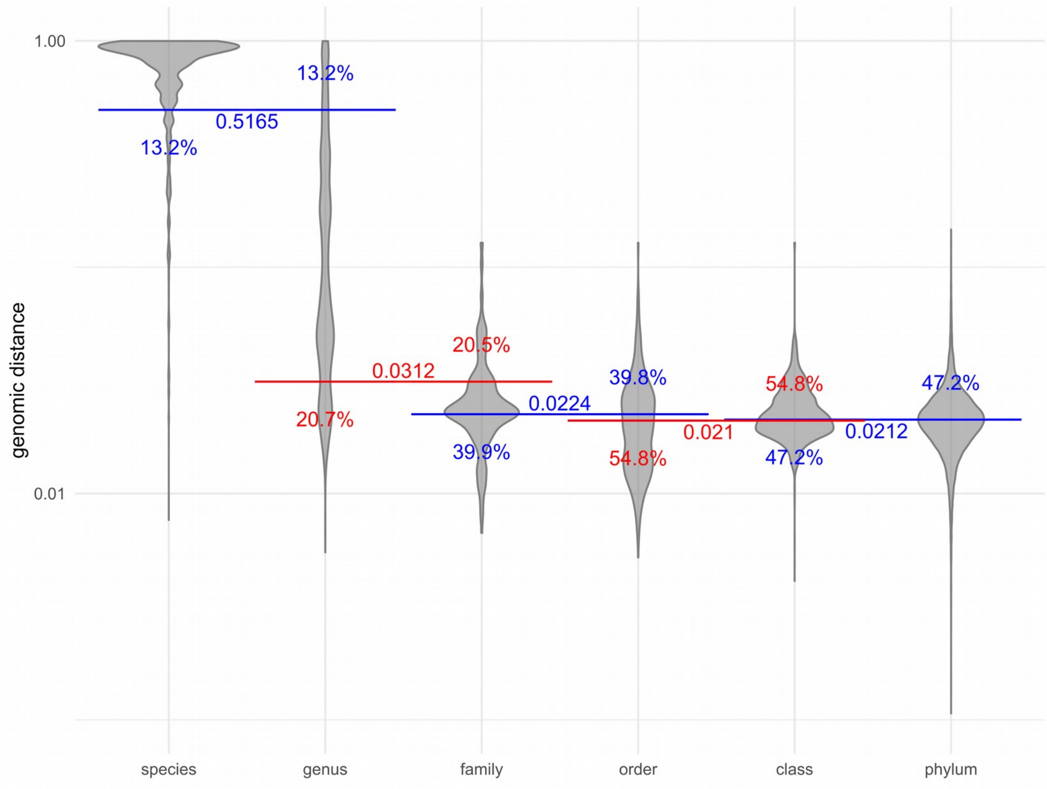

Supplemental Figure S3. Pairwise genomic distances between all genomes of various species, calculated by Dashing with k-mer size 16. Distances are shown as box-and-whiskers plots, with outliers marked as dots and mean distances per species marked with crosses. The red vertical line shows the threshold distance between the ranks, calculated to minimize the share of genomic distances on the wrong side of the line (misclassified distances). Only species with at least 20 pairwise distances are shown.

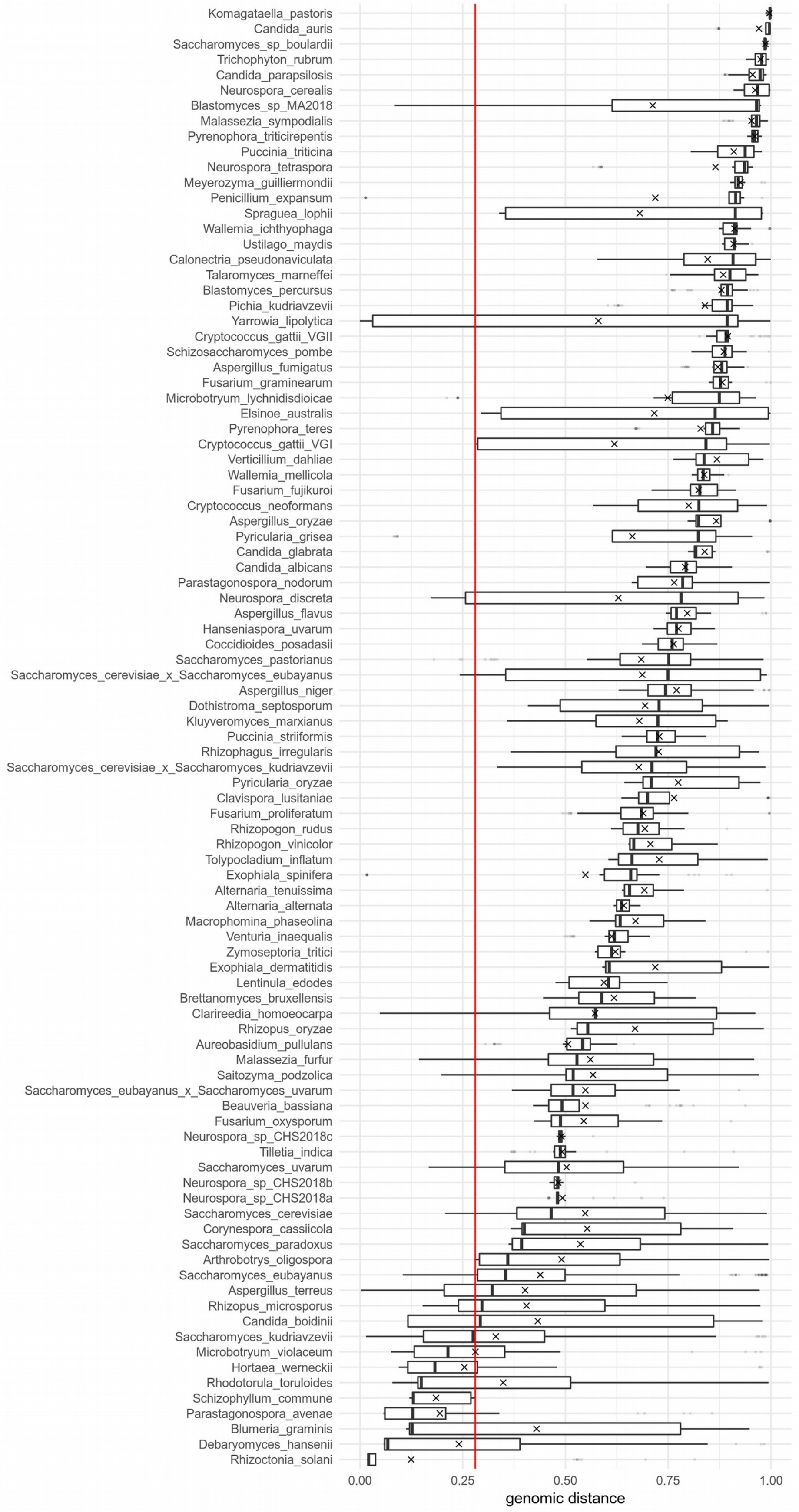

Supplemental Figure S4. Pairwise genomic distances within fungal families, orders and classes, calculated by Mash with k-mer size 16, ordered by their median genomic distances. Distances are shown as box-and-whiskers plots, with outliers marked as dots and mean distances per genus marked with crosses. Only genera with at least 20 pairwise distances are shown.

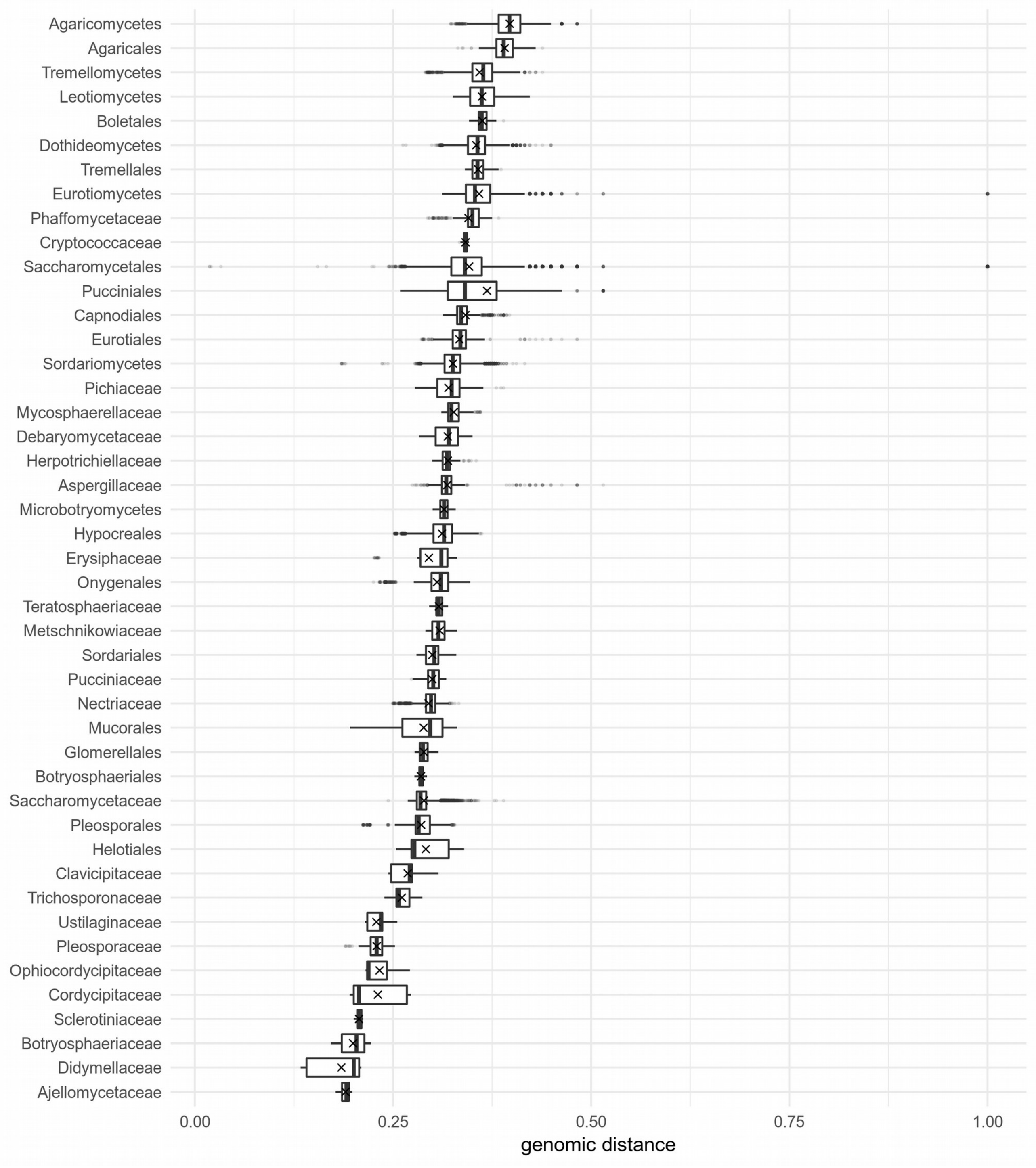

Supplemental Table S1. p-values for post-hoc pairwise estimated marginal means test between pairwise genomic distances at different taxonomic levels. The values are provided for all three methods of genomic distance estimation and both used dataset (full dataset – GenBank, and reduced dataset – RefSeq) – see Methods for details.

	RefSeq			GenBank	
	Dashing	Mash	Shared k-mers	Dashing	Mash
**species vs genus**	**<.0001**	**<.0001**	**<.0001**	**<.0001**	**<.0001**
**species vs family**	**<.0001**	**<.0001**	**<.0001**	**<.0001**	**<.0001**
**species vs order**	**<.0001**	**<.0001**	**<.0001**	**<.0001**	**<.0001**
**species vs class**	**<.0001**	**<.0001**	**<.0001**	**<.0001**	**<.0001**
**species vs phylum**	**<.0001**	**<.0001**	**<.0001**	**<.0001**	**<.0001**
**genus vs family**	**<.0001**	**<.0001**	**<.0001**	**<.0001**	**<.0001**
**genus vs order**	**<.0001**	**<.0001**	**<.0001**	**<.0001**	**<.0001**
**genus vs class**	**<.0001**	**<.0001**	**<.0001**	**<.0001**	**<.0001**
**genus vs phylum**	**<.0001**	**<.0001**	**<.0001**	**<.0001**	**<.0001**
**family vs order**	**0.0001**	**<.0001**	**<.0001**	**<.0001**	**<.0001**
**family vs class**	0.4466	**<.0001**	**0.0005**	0.7579	**<.0001**
**family vs phylum**	**<.0001**	**<.0001**	**<.0001**	**<.0001**	**<.0001**
**order vs class**	**0.0002**	**<.0001**	0.4817	**<.0001**	**<.0001**
**order vs phylum**	**<.0001**	**<.0001**	0.9936	**<.0001**	**<.0001**
**class vs phylum**	**<.0001**	**<.0001**	**0.0057**	**<.0001**	**<.0001**
